# Personality and Cognitive Functions in Violent Offenders – Implications of Character Maturity?

**DOI:** 10.3389/fpsyg.2020.00058

**Published:** 2020-01-28

**Authors:** Helena Seidl, Thomas Nilsson, Björn Hofvander, Eva Billstedt, Märta Wallinius

**Affiliations:** ^1^Region Sormland, Katrineholm, Sweden; ^2^Centre for Ethics, Law and Mental Health, Institute of Neuroscience and Physiology, University of Gothenburg, Gothenburg, Sweden; ^3^Department of Forensic Psychiatry, National Board of Forensic Medicine, Gothenburg, Sweden; ^4^Department of Clinical Sciences Lund, Child and Adolescent Psychiatry Research Unit, Lund University, Lund, Sweden; ^5^Gillberg Neuropsychiatry Centre, Institute of Neuroscience and Physiology, University of Gothenburg, Gothenburg, Sweden; ^6^Research Department, Regional Forensic Psychiatric Clinic, Växjö, Sweden

**Keywords:** cognitive functions, intelligence, executive functions, offenders, personality, character maturity, prison

## Abstract

Previous research has suggested that personality and cognitive functions are essential in the emergence of persistent aggressive antisocial behaviors and that character maturity could be an important protective factor against these behaviors. The aims of this study were (1) to determine associations between personality traits, intellectual ability, and executive function in young male violent offenders, and (2) to investigate differences in intellectual ability and executive function between groups of violent offenders with low, medium, and high character maturity. A cohort of 148 male violent offenders (18–25 years of age) participated in this study. The Temperament and Character Inventory was used as a self-report measure of personality traits, and cognitive functions were measured with the Wechsler Adult Intelligence Scale – Third Edition and the Cambridge Neuropsychological Test Automated Battery. Intellectual ability was negatively correlated to the temperament dimension Harm Avoidance and the character dimension Self-Transcendence, and positively correlated to the character dimensions Self-Directedness and Cooperativeness and the temperament dimension Novelty Seeking. Visual sustained attention correlated positively to the temperament dimension Persistence and negatively to the temperament dimension Harm Avoidance. Spatial working memory correlated negatively to the character dimension Cooperativeness. Character maturity, however, did not affect intellectual and executive functions to a statistically significant degree. Our findings indicate that offender personality characteristics such as optimism, responsibility, empathy, curiosity, and industry that would seem more favorable to positive intervention outcomes are related to better cognitive functioning. Possible implications are that interventions in offender populations could be more effective if tailored to participants’ personality dimensions and cognitive proficiencies, rather than offered as “one size fits all.”

## Introduction

Interpersonal violence is one of the leading causes of death worldwide, especially for individuals aged 15–44 years, and it is recognized as one of the most important public health problems causing, apart from premature death, injury, disability, and impaired quality of life for millions of people (Krug et al., 2002). Interpersonal violence is also associated with tremendous costs for society as a whole, including health care, legal costs, productivity loss, and human suffering (Corso et al., 2007).

Evidence shows that a small and pathological subgroup of individuals is responsible for a disproportionate amount of crime and violence. Falk et al. (2014) found that the majority of violent crimes in a Swedish population was committed by a small number of persistent violent offenders, typically male and defined by an early onset of violent criminality. Indeed, the age-crime curve describes a clear increase in crime rates in mid-adolescence, peaking at about age 17, followed by a steep decline in young adulthood (Moffitt, 1993; Blonigen, 2010). However, data on self-reported deviant behavior and delinquency show a sharp increase in antisocial behaviors during childhood as well (Farrington et al., 2009). Better knowledge of the characteristics of this particular group is needed to develop specific treatment interventions and preventive strategies.

Different developmental trajectories have been suggested to explain the origins of aggressive antisocial behaviors including violent offending, comprising early starters: childhood-limited antisocial individuals, life-course persistent (LCP) violent offenders, and late-onset: adolescence-limited (AL) offenders, and adult onset offenders (Moffitt, 1993; Raine et al., 2005). Individuals on the LCP pathway of antisocial behavior have the greatest impact on society and on their own mental and physical health outcomes as adults (Odgers et al., 2007). Previous research has shown considerable continuity in antisocial behaviors over time; these individuals who become persistent offenders not only start early but also display exceptionally frequent antisocial behaviors (Loeber, 1982).

A large body of research describes the characteristics of persistent offenders, including their personality traits and intellectual and neuropsychological functioning. Environmental features, such as social and familial factors, associated with future violence have also been identified (Maas et al., 2008; Frisell et al., 2012). According to LCP theory, subtle variations in a child’s temperament, behavioral development, and neuropsychological/cognitive abilities interact with suboptimal parenting and a maladaptive social environment, further exacerbating the individual’s antisocial behavior (Moffitt and Caspi, 2001). Over the course of time this interplay progresses into a disordered personality with hallmark aggressive antisocial behaviors that persist well into adulthood (Moffitt et al., 2002). Thus, there is a continuous process over time of reciprocal interactions between individual traits and environmental factors that set the stage for future violent offending. These children, who later become delinquent, display early conduct problems, including lying, stealing, and truancy (Loeber and Dishion, 1983) and physical aggression (Broidy et al., 2003), and the frequency of these behaviors has been associated with persistent offending and unsuccessful outcomes in adulthood (Farrington et al., 2009). Poor educational achievement has also been linked to delinquency in males (Loeber and Dishion, 1983), and severe school adjustment problems such as bullying, early onset of truancy, and early school leaving have been associated with high levels of aggressive antisocial behaviors in young male violent offenders (Wallinius et al., 2016). Recently, a new model integrating psychological and social factors, the integrated psychosocial model of criminal social identity (IPM-CSI; Boduszek et al., 2016; Spink et al., 2019), has been suggested to aid the understanding of development of persistent criminality. The IPM-CSI considers previous research on antisocial development and describes the interplay between psychosocial factors, e.g., exposure to an antisocial environment, identity crisis, a need for identification with a criminal group, and personality traits, and suggests how an interplay between these factors contributes to the development of a criminal social identity. Obviously, there is a need to expand the current models and take interaction between factors known to increase the risk of aggressive antisocial behaviors into account.

It has recently been argued that personality traits are important predictors of criminality, even more so than socio-economic variables (O’Riordan and O’Connell, 2014). Early onset behavioral patterns have been demonstrated to be related to personality traits in children assessed at ages 3 and 18 (Caspi and Silva, 1995), showing the highest scores on aggression at age 18 in children identified as “undercontrolled” at age 3. Furthermore, these temperamental qualities at age 3 were shown to predict behavior problems, personality structure, quality of interpersonal relationships, mental health problems, and criminal behavior in young adulthood (Caspi, 2000). Persistent violent offenders are often diagnosed with antisocial personality disorder and/or psychopathy or psychopathic traits. In a meta-analytic review, Miller and Lynam (2001) found support for an association between antisocial behavior and structural models of personality when examining, among others, Cloninger’s seven-factor temperament and character model (Cloninger et al., 1993; Cloninger, 1994). Several studies have investigated dimensions of temperament and character in juvenile offenders with conduct disorder (Lennox and Dolan, 2014), young adults with antisocial personality disorder (Basoglu et al., 2011), and persistent offenders with psychopathic traits (Snowden and Gray, 2010), using the Temperament and Character Inventory (TCI), which is based on Cloninger’s psychobiological model (Cloninger et al., 1993).

Cloninger’s psychobiological model of the structure and development of personality comprises two major domains of personality: temperament, including four dimensions, and character, including three dimensions; the distinctions between them seem to parallel major neural systems for procedural versus propositional memory and learning (Cloninger, 1994). The temperament dimensions involve the neural system for procedural memory and learning, where temperament traits become manifest early in life, are developmentally stable, and remain uninfluenced by sociocultural circumstances, reflecting basic emotional drives and automatic responses to emotional stimuli. The character dimensions reflect concept-based goals and values and involve the neural system of propositional memory and learning. Character traits mature gradually from childhood into late adulthood and reflect differences in higher cognitive processes underlying the goals and values of the individual. According to the model, the temperament domain consists of four dimensions; Harm Avoidance (HA): a bias to inhibit behavior in response to adverse stimuli; Novelty Seeking (NS): a tendency to initiate exploratory behavior in response to novelty; Reward Dependence (RD): a bias to respond strongly to social approval; and Persistence (PS): a tendency to persevere despite frustration or fatigue. The character domain comprises three dimensions; Self-Directedness (SD): the extent to which the individual is responsible, goal-oriented, and able to adapt their behavior accordingly; Cooperativeness (CO): the extent to which the individual perceives themselves as part of the community and expresses empathy, tolerance, and compassion to other people; and Self-Transcendence (ST): the extent to which the individual conceives a higher meaning and sees themselves as integrated with the existence at large. Together, the character dimensions SD and CO form the concept of character maturity, in which high SD has been strongly related to both wellness and happiness and high CO to wellness in healthy middle-aged individuals (Cloninger and Zohar, 2011). In contrast, extreme immaturity has been associated with the presence of a personality disorder. Previous research suggests that the diagnosis of a personality disorder is based on character maturity, while the various subtypes of personality disorders are differentiated by temperament dimensions (Svrakic et al., 2002; Richter and Brändström, 2009).

In addition to specific personality traits, a wealth of evidence supports the proposition that persistent offenders also display neurocognitive impairments that may play an important role in the emergence of aggressive antisocial behaviors, such as reduced intellectual ability, usually measured by Wechsler Scales for instance the Wechsler Adult Intelligence Scale (WAIS) (Wechsler, 1997) and executive dysfunctions, measured, for example, by the Cambridge Neuropsychological Test Automated Battery (CANTAB; Cambridge Cognition Ltd., Cambridge, United Kingdom), which is frequently used in both clinical samples and healthy controls (e.g., Robbins et al., 1998; Torgersen et al., 2012).

It has consistently been shown that antisocial groups score lower than controls on verbal IQ and higher on performance IQ across all ages, with the largest effect sizes for adolescents (Isen, 2010). IQ has also been associated with level of delinquency in a stepwise manner, with non-delinquent boys performing better on both verbal and full-scale IQ than serious delinquents, and moderately delinquent boys performed between the two extremes (Lynam et al., 1993). The IQ-delinquency relation was robust and independent of race, social class, motivation, and impulsivity. Antisocial adults have been found to exhibit broad and pervasive impairments in various executive functions (Hancock et al., 2010), including attentional set-shifting (Bergvall et al., 2001), response inhibition (Meijers et al., 2017), and planning and visual memory (Dolan and Park, 2002); the relation between antisocial behaviors and executive deficits has been found to be robust and statistically significant in meta-analyses (Ogilvie et al., 2011) with medium to large effect sizes (Morgan and Lilienfeld, 2000).

Despite the knowledge that both personality traits and cognitive functions are important in understanding aggressive antisocial behaviors, little research has investigated the association between personality and cognitive and executive functions in offenders (Rasmussen et al., 2001). This is remarkable, especially since executive functions and personality dimensions can be viewed as sharing one neural ground, the prefrontal cortex (PFC). Indeed, lesion studies have repeatedly and over time demonstrated that prominent personality alterations frequently follow damage to the PFC (Stuss and Benson, 1984; Barrash et al., 2011, 2018; Schneider and Koenigs, 2017), and neuroimaging studies have reported that different areas of the PFC are compromised both structurally and functionally in antisocial, violent, and psychopathic populations (Raine and Yang, 2006; Yang and Raine, 2009; Gregory et al., 2012), in both adolescents and adults (Umbach et al., 2015).

The few exceptions to study both cognitive functions and personality traits as measured by the TCI have demonstrated that offenders with personality disorders score low on SD and CO and make more errors on an attentional set-shifting task (Bergvall et al., 2003). However, the personality disordered offenders did not differ from the comparison groups on measures of visual working memory and planning. In a population-based sample of 15-year-old twins, discrepancies showed small relationships between cognitive ability and both SD and ST, although no significant relationships between character and cognitive ability were demonstrated (Mousavi et al., 2015). A recent study on conduct disordered children suggests that character maturity is an important protective factor against aggressive and antisocial behavior (Kerekes et al., 2017); this has also been demonstrated in adult prison inmates (Falk et al., 2017). In further exploring the association between character maturity and antisocial behavior, Nilsson et al. (2016) divided violent offenders into three different groups based on level of character maturity (low, medium, and high) and found that higher levels of character maturity appeared to be associated with less aggressive antisocial behaviors, even when controlling for ADHD. Also, the lower the maturity level, the more externalizing disorders (ADHD, conduct disorder, and aggressive antisocial behaviors) were present in the offenders. Thus, better understanding of the contributions of character maturity and cognitive functioning to aggressive antisocial behaviors may have important implications for treatment and rehabilitation interventions in correctional settings.

In response to the identified needs of better knowledge in the field, this study was aimed to explore the extent to which personality and cognitive functions (intellectual ability, executive functions) are associated in young male violent offenders, through two specific aims:

1. Determine associations between personality (as measured by TCI) and cognitive functions (as measured by WAIS and CANTAB).
2. Investigate differences in cognitive functions between groups of violent offenders with low, medium, and high levels of character maturity.

## Materials and Methods

### Participants

Participants in this study (*n* = 148) were drawn from a Swedish prison study of young male offenders convicted of physically violent and/or sexual criminality, the Development of Aggressive Antisocial Behavior Study (DAABS). The DAABS included young male offenders serving a prison sentence at a facility in the Western region of the Swedish Prison and Probation Services between March 2010 and July 2012. All male inmates aged 18–25 were invited to participate in the study; inmates with insufficient language skills and those who were relocated before the data could be safely collected were excluded. The participation rate was 71% of all who met inclusion criteria, and in-depth descriptions of the total DAABS cohort (*n* = 269) are available in previous publications (e.g., Wallinius et al., 2016; Billstedt et al., 2017; Hofvander et al., 2017). In the total DAABS cohort, the majority of the offenders had a non-sexual violent history, with only *n* = 31 offenders reporting an offense history of sexual offenses (Wallinius et al., 2016). From all the participants in the DAABS, 148 who provided valid TCI protocols, 147 who provided valid WAIS-III protocols, and 119–133 who provided valid CANTAB protocols were included in the current study.

### Instruments

#### Temperament and Character Inventory

Personality was assessed using the TCI (Cloninger, 1994), based on Cloninger’s psychobiological model of personality (Cloninger et al., 1993). The TCI consists of 238 yes/no items measuring four temperament dimensions (NS, HA, RD, and P) and three character dimensions (SD, CO, ST). *T*-scores were calculated for each dimension based on Swedish normative data (Brändström et al., 1998), and the two character dimensions of SD (responsibility and goal-directed behavior) and CO (empathy and compassion) were used to identify character maturity and to divide the study group into three distinct groups; low, medium, and high character maturity (HCM). The low character maturity (LCM) group scored more than 1 SD below the mean for both character dimensions and included 43 male offenders with a mean age of 22.1 years (*SD* = 1.9). The medium character maturity (MCM) group comprised offenders (*n* = 54) in whom one of the two character dimensions was 1 SD below the mean and the other dimension within the normal range. The MCM group had a mean age of 21.7 years (*SD* = 1.9). The HCM group consisted of those with scores within or above the normal range for both character dimensions, including 51 male offenders with a mean age of 21.6 years (*SD* = 1.9).

#### Wechsler Adult Intelligence Scale-Third Edition

General intellectual functioning was assessed on the General Ability Index (GAI, Tulsky et al., 2001) of the Wechsler Adult Intelligence Scale-Third Edition (WAIS-III; Wechsler, 1997), including the Verbal Comprehension Index (VCI) and the Perceptual Organization Index (POI). The GAI is a global score based on the sum of scaled scores on the subtests that make up the VCI and POI, and it provides an overall estimate of general intellectual ability. The VCI consists of the verbal subtests Vocabulary, Similarities, and Information, and measures crystallized intelligence, whereas the POI comprises the non-verbal subtests Matrix Reasoning, Block Design, and Picture Completion, and measures fluid intelligence. Descriptive statistics are available in Table 2. In the current study, the WAIS GAI, VCI, and POI mean scores did not differ significantly from the corresponding WAIS mean scores in the total DAABS cohort when tested using Student’s *t*-test (data provided upon request).

#### Cambridge Neuropsychological Test Automated Battery

Subtests from the CANTAB were used to assess five different executive functions: cognitive flexibility, planning and problem-solving, working memory, response inhibition, and attention. The CANTAB is a computerized neuropsychological test battery that has previously been used in research on antisocial and violent behavior (Bergvall et al., 2001; Dolan and Park, 2002).

The Intra-Extra Dimensional Set Shift (IED), a computerized analog of the Wisconsin Card Sorting Test, featuring visual discrimination and attentional set formation, maintenance, shifting and flexibility of attention, was used to assess cognitive flexibility. Participants were presented with two distinct types of stimuli: color-filled shapes and white lines. They were then instructed to find out which stimulus was correct by trial and error. Feedback allowed the participants to learn the underlying rule. Outcome measures used were the number of stages reached (IED stages completed), the total number of errors made (IED total errors adjusted) and errors made at set-shifting (IED EDS errors). The IED median scores among the participants in the current study did not differ significantly from the corresponding IED median scores in the total DAABS cohort when tested with Mann–Whitney *U* test (data provided upon request).

The Stockings of Cambridge (SOC) is a computerized version of the Tower of London task, assessing the participants’ ability to engage in spatial planning and problem solving. Participants were instructed to move colored balls in the lower part of the screen to copy the pattern of balls presented in the upper part, using as few moves as possible. Outcome measures used were the mean initial thinking time before attempting to solve a five-move problem (SOC MITT5; measured in milliseconds), the mean subsequent thinking time (SOC MSTT5; measured in milliseconds), and the number of problems solved in the minimum number of moves (SOC PS). The median scores for the SOC measures did not differ significantly between the participants in the current study and the total DAABS cohort when tested with Mann–Whitney *U* test, except for SOC MSTT5 (*p* = 0.041; data provided upon request) where the participants in the current study had lower reaction times.

The Spatial Working Memory (SWM) requires retention and manipulation of visuospatial information; it also assesses heuristic strategy related to working memory. Several colored boxes were presented on the screen, and the participants were required to find a hidden token by using the process of elimination. All boxes contained a token only once, i.e., participants had to remember in which box they had already found one. The number of boxes was gradually increased to eight. Outcome measures used were SWM between errors (a summed measurement for all trials of four or more tokens), as an indicator of working memory, and SWM strategy, as a measure of optimal strategy. The SWM median scores among the participants in the current study did not differ significantly from the corresponding SWM median scores in the total DAABS cohort when tested with Mann–Whitney *U* test (data provided upon request).

The Stop-Signal Task (SST) assesses the participants’ ability to inhibit a response when given auditory feedback and was used as a measure of response inhibition in this study. The test consists of two parts: in the first, the participants were told to press the left hand button when they saw a left-pointing arrow and the right hand button when they saw a right-pointing arrow. In the second part, the participants were told to continue pressing the buttons when presented with the arrows, but to withhold their response if they heard an auditory signal (a beep). The main outcome measure was the stop signal reaction time (SST SSRT), which is a covert measure of inhibitory control, measured in milliseconds. The SST SSRT median scores in the current study sample were significantly lower than those in the total DAABS cohort in a Mann–Whitney *U* test (*p* = 0.030), with lower reaction times for the participants in the current study (data provided upon request).

The Rapid Visual Information Processing (RVP) is a sensitive measure of visual sustained attention. The participants were required to detect target sequences of three digits each among serially appearing digits. Outcome measures used were RVP A’, which reflects the participant’s accuracy at detecting a target sequence of numbers, and RVP mean latency (measured in milliseconds) as an indicator of sustained attentional function. Both outcome measures differed significantly between the current study sample and the total DAABS cohort in Mann–Whitney *U* tests, with higher RVP A’ scores (*p* = 0.027) and lower RVP mean latency scores (*p* ≤ 0.0001) (data provided upon request).

### Data Analysis

Data were anonymized and analyzed using IBM SPSS Statistics 22. Two-tailed *p*-values and a significance threshold of *p* < 0.05 were used due to the explorative design of the study. All data were assessed for normality using plots and statistics based on the distribution of data. Correlations between cognitive measures and personality measures (Aim 1) were investigated using Pearson’s *r* or Spearman’s rho (*r*_*s*_). Differences in cognitive functions for offenders with different levels of character maturity (Aim 2) were investigated by a one-way between-groups multivariate analysis of variance (MANOVA). Level of character maturity was used as independent variable, while the following four variables that showed significant associations with any of the personality dimensions were used as dependent variables: WAIS VCI, WAIS POI, RVP A’, and SWM strategy. Basic assumption testing was conducted for univariate and multivariate outliers, normality, linearity, homogeneity of variance-covariance matrices, and multicollinearity, with no serious violations except for two outliers were identified in the univariate and multivariate analyses and removed from the multivariate analysis, making a total of 145 offenders available for the MANOVA. Pillai’s Trace was used as the multivariate significance test due to unequal number of individuals in the three groups of character maturity.

## Results

Descriptive statistics on the TCI personality dimensions and the CANTAB measures are provided in Tables 1, 2.

**TABLE 1:** *T*-scores for temperament and character dimensions in violent offenders (*n* = 148).

**TCI-dimension**	**Minimum**	**Maximum**	**Mean**	**SD**
	**(*T*-scores)**	**(*T*-scores)**	**(*T*-scores)**	**(*T*-scores)**
Novelty Seeking	28	71	54.89	8.82
Harm Avoidance	29	88	52.59	12.13
Reward Dependence	24	68	45.10	9.31
Persistence	27	68	47.61	9.45
Self-Directedness	14	68	43.28	13.19
Cooperativeness	5	67	37.91	12.14
Self-Transcendence	31	89	56.58	12.57

*TCI, Temperament and Character Inventory.*

**TABLE 2:** General intellectual functioning (WAIS-III) and executive functions (CANTAB) in violent offenders.

	***n***	**Minimum**	**Maximum**	**Mean**	**SD**
**WAIS-III (composite scores)**					
General Ability Index	147	67	126	93.78	10.78
Verbal Comprehension Index	147	65	114	90.53	10.49
Perceptual Organization Index	147	62	130	98.97	14.17
**CANTAB (scores)**					
IED EDS errors	133	0	34	14.27	10.07
IED Stages completed	133	1	9	8.09	1.23
IED Total errors adjusted	133	7	211	37.18	28.28
SOC Mean initial thinking time (ms)	132	0	29379	5936.73	4722.05
SOC Mean subsequent thinking time (ms)	132	0	8330	1062.69	1535.53
SOC Problems solved in minimum moves	133	2	12	8.32	1.80
SWM Between errors	133	0	114	22.69	17.36
SWM Strategy	133	0	43	32.53	5.18
RVP A’	119	1	1	0.89	0.05
RVP Mean latency (ms)	119	233	947	400.84	106.44
SST SSRT (ms)	123	80	584	185.83	72.02

*WAIS-III, Wechsler Adult Intelligence Scale-Third Edition; CANTAB, Cambridge Neuropsychological Test Automated Battery; IED, Intra-Extra Dimensional set shift; EDS, Errors made at Set-shifting; SOC, Stockings of Cambridge; SWM, Spatial Working Memory; RVP A’, Rapid Visual Information Processing Accuracy; SST SSRT, Stop-Signal Task Stop Signal Reaction Time.*

### Associations Between Personality Dimensions and Cognitive Functions

Table 3 shows correlations (Pearson’s *r*) between WAIS-III scores and temperament and character *T*-scores from the TCI. For intellectual functioning, the GAI was negatively correlated with the temperament dimension HA and positively correlated with the character dimensions SD and CO. For the VCI, a positive correlation to the temperament dimension NS was observed, and a negative correlation was found for the character dimension ST. Finally, the POI was negatively correlated with the temperament dimension HA but positively correlated with the character dimensions SD and CO. All effect sizes, as demonstrated by the *r*-values in Table 3, were small.

**TABLE 3:** Correlations between general intellectual functioning (WAIS-III) and temperament and character dimensions (TCI) in violent offenders (*n* = 148).

**TCI**																					
	**Temperament**												**Character**								
	**NS**			**HA**			**RD**			**PS**			**SD**			**CO**			**ST**		
	***r***	***p***	***r*^2^**	***r***	***p***	***r*^2^**	***r***	***p***	***r*^2^**	***r***	***p***	***r*^2^**	***r***	***p***	***r*^2^**	***r***	***p***	***r*^2^**	***r***	***p***	***r*^2^**
WAIS-III (composite scores)																					
General Ability Index	0.15	ns		−0.21	0.011	0.04	−0.05	ns		−0.07	ns		0.18	0.032	0.03	0.16	0.050	0.03	−0.14	ns	
Verbal Comprehension Index	0.23	0.005	0.05	−0.16	ns		−0.12	ns		−0.03	ns		0.06	ns		0.01	ns		−0.18	0.030	0.03
Perceptual Organization Index	0.06	ns		−0.20	0.013	0.04	0.03	ns		−0.07	ns		0.22	0.007	0.05	0.22	0.007	0.05	−0.08	ns	

*WAIS-III, Wechsler Adult Intelligence Scale-Third Edition; TCI, Temperament and Character Inventory; NS, Novelty Seeking; HA, Harm Avoidance; RD, Reward Dependence; PS, Persistence; SD, Self-Directedness; CO, Cooperativeness; ST, Self-Transcendence.*

The relationships between temperament, character, and executive functions were investigated using Spearman’s rho. A negative correlation between visual sustained attention and the temperament dimension HA was found (*r*_*s*_ = −0.22, *p* = 0.016), while visual sustained attention correlated positively with the temperament dimension PS (*r*_*s*_ = 0.19, *p* = 0.039). Spatial working memory correlated negatively with the character dimension CO regarding both retaining spatial information (*r*_*s*_ = −0.17, *p* = 0.049), and optimal strategy (*r*_*s*_ = −0.18, *p* = 0.036). All effect sizes, as demonstrated by the *r*_*s*_-values, were small.

### Differences in Cognitive Functions Between Groups With Different Levels of Character Maturity

When the two general intelligence functions of WAIS (VCI, POI) and the two executive functions RVP A’ and SWM strategy were tested in a MANOVA, no significant effect for level of character maturity was found: *F*(8,224) = 1.49, *p* = 0.16; Pillai’s Trace = 0.10; partial eta squared = 0.05.

## Discussion

This study explored associations between personality dimensions and cognitive functions, as well as differences in cognitive function between groups with different levels of character maturity in a nationally representative Swedish cohort of young male violent offenders. Our main findings were that intellectual ability was associated, yet with small effect sizes, with personality traits such as NS, HA, SD, CO, and ST, whereas executive functions were associated with HA, PS, and CO. However, when tested in a multivariate analysis, level of character maturity did not significantly affect the cognitive measures. All findings should be considered as explorative.

When investigating personality traits in relation to intellectual ability we found that participants high in general intellectual ability were low in HA (usually associated with being courageous, energetic, and outgoing), high in SD (such as being responsible, resourceful, and goal-oriented), and high in CO (being empathic, compassionate, and principled). This seems reasonable, since intellectual ability in or above the normal range usually implies sufficient or increased ability to deal with whatever challenges life may present, which may be related to these personality dimensions. This positive outlook on life could, over time, in interaction with favorable factors in the environment lead to the development of responsibility, reliability, and self-confidence, as well as empathy, tolerance, and compassion, that is, to adequate and satisfactory development toward a mature personality. However, these speculations would need further investigations in future research. In considering intervention strategies, it might be more likely for individuals with these favorable prerequisites to benefit more from treatment interventions and thus have more promising outcomes than their less fortunate peers. A probable consequence of this kind of finding is to encourage more focus on offenders with less cognitive and personality resources through the design and provision of better tailored and more extensive interventions.

Individuals high in verbal ability were also high in NS (usually associated with being quick-tempered, curious, and impulsive), but low in ST (less spiritual and humble). Like general intellectual ability, verbal intellectual ability in or above the normal range could be assumed to correspond with being curious, quick-tempered, and easily bored with an increased ability and willingness to explore novel situations. This might also be interpreted to signal psychopathic traits, which have been associated with higher levels of NS and lower levels of CO, HA, and RD (Snowden and Gray, 2010). However, the prevalence of psychopathic traits in the DAABS cohort has been reported as somewhat lower than what might be expected in a sample of violent offenders (Delfin et al., 2018), possibly due to the young age of the offenders, whose psychopathic traits may not yet be fully established. Thus, we can assume that the associations between verbal IQ, temperament, and character cannot be entirely accounted for by psychopathic traits. The low propensity for spirituality and humbleness could be explained by the Swedish society’s tendency to hold secular-rational values, which could also be reflective of the study population since a majority was born in Sweden (Wallinius et al., 2016). Furthermore, as described by Cloninger et al. (1993), ST is considered a developmental process and thus potentially increases with age, therefore the participants in this study might be too young for their spirituality and fulfillment in life to have developed. An alternative interpretation is that low ST could reflect a low sense of coherence in which the individuals experience themselves and their relation to the outside world as less comprehensible, manageable, and meaningful.

Individuals high in perceptual ability were low in HA, high in SD, and high in CO. This is in line with the result for general intellectual ability described above, and it seems conceivable that perceptual ability is the factor that constitutes the result for intellectual ability as a whole. That we were not able to demonstrate the corresponding result for verbal ability is somewhat unforeseen and not in line with previous research demonstrating a stable association between IQ and delinquency. In other studies, lower *verbal* IQ is consistently associated with antisocial behavior (Isen, 2010), which in turn is associated with low SD and low CO in violent offenders (Falk et al., 2017). In the present study group, however, it would seem that verbal intellectual ability is not an optimal outcome measure, which is surprising since poor educational achievement is also associated with antisocial behavior (Loeber and Dishion, 1983) and the relationship between verbal ability and educational achievement has been found to be strong as it is for perceptual ability (Roth et al., 2015) or even stronger (Colom and Flores-Mendoza, 2007). It should also be noted that the current study sample did not differ from the total DAABS cohort in general intellectual abilities, leading to the conclusion that our findings on this part were not affected by better general intellectual functioning in the participants who provided valid TCI protocols.

When we investigated personality traits in relation to executive functions, we found that individuals high in visual sustained attention were low in HA and high in PS (such as being industrious, perseverant, and ambitious). Individuals who were able to sustain visual attention over time tended to be more confident, energetic, and optimistic, and displayed a greater ability to work hard, persevere through frustration and fatigue, and to intensify their effort for long term rewards. Individuals high in spatial working memory, in both retaining information and using efficient strategy, were low in CO. Given the nature of aggressive antisocial behaviors, it seems plausible that the individuals in this study generally display less empathy, tolerance, and compassion, and that they are less likely to consider themselves integrated with society, as reported in previous research (Bergvall et al., 2003; Falk et al., 2017). Therefore, one feasible interpretation of these findings could be that these personality traits are pervasive and profound in these individuals to the extent that they are manifest in the majority, including those individuals whose executive functions remain intact. However, it should be noted that the current study sample differed from the total DAABS cohort in shorter overall reaction times and better visual sustained attention. This would actually seem logical, since completion of the TCI protocol requires perseverance and sustained vigilance that could be expected to be lowered in the total DAABS cohort due to the high prevalence of ADHD (see Billstedt et al., 2017). That is, our results may have been affected by somewhat better executive functions in perseverance, cognitive processing speed, and sustained attention in our study sample. This needs to be considered in designing future studies on similar groups, where the inclusion of measures requiring moderate or high levels of perseverance and sustained attention likely will lead to attrition.

We found no significant results in our multivariate analysis of the possible effect of different levels of character maturity on cognitive functions, either separately or as a whole. Thus, although cognitive measures and personality dimensions were (weakly) associated on a bivariate level in this study group, this pattern did not hold when employing a higher-order analysis using a composite measure of cognitive functions. A possible conclusion is that character maturity may not affect intellectual and executive functions to such a degree that it is either statistically or clinically relevant. However, since this is, to the best of our knowledge, the first investigation of these relationships, replications are needed before any firm conclusions can be drawn.

Several limitations need consideration in this study. First, our findings may not be generalizable to populations outside of Swedish young male violent offenders. We also have indications that the current study sample was characterized by somewhat better executive functions in the areas of visual sustained attention and cognitive processing speed than the total DAABS cohort. Since we did not have access to a comparison group, our findings should not be generalized to other groups. Second, a distinction has been proposed between cool executive functions, such as attention, planning, and working memory, and hot executive functions that engage emotion regulation and motivation, such as affective decision-making. Previous research has shown an association between impairment in affect and motivation in conduct disordered children (Rubia, 2011) and impairments in hot executive functions in violent offenders (De Brito et al., 2013). In this study, due to design considerations, we did not include any measures of hot executive functions and thus had no opportunity to study potential associations with hot executive functions. Third, since the present study group is a subgroup of a larger cohort (e.g., Hofvander et al., 2017), it is possible that the participants might represent different developmental trajectories of aggressive antisocial behaviors, affecting their levels of cognitive impairment and personality deviations. Since the participants in both the total DAABS cohort and in the current subgroup are still young, we have no abilities at the moment to determine different developmental trajectories to control for this; however, we did report differences between the study subgroup and the total DAABS cohort. Fourth, the method for choosing dependent variables for the MANOVA from significant results from the bivariate analyses can be criticized, since the choice of dependent variables should be made based on theoretical assumptions. In this case, there was very limited literature available for theoretical assumptions to be stated clearly, which is why we chose the current method. The reason for dividing character maturity in three groups and applying MANOVA instead of using character maturity as a continuous measure and applying correlation analyses was that the sparse literature that exist on this matter has divided character maturity in low, medium, and high levels, and our results can thus be used for continued research with hypothesis testing. Fifth, due to the lack of previous findings to base hypotheses on, the current study could only apply an explorative design. Finally, since no female offenders were included in this study, our findings are applicable to males only.

## Conclusion

Previous research has demonstrated associations between aggressive antisocial behaviors and personality traits and cognitive functions. In this study, however, the associations between these variables were few and weak and did not hold in a multivariate analysis. Contrary to previous research reporting a consistent pattern of associations between levels of character maturity, externalizing disorders such as ADHD, conduct disorder, and substance use disorder, and aggressive antisocial behaviors (Nilsson et al., 2016), we were not able to demonstrate any associations between personality traits, cognitive functions, and levels of character maturity in the same study group. In order to understand our findings, the specific limitations of this study need to be considered. Furthermore, since the area of associations between cognitive functions and personality dimensions is relatively under-researched, continued research on this subject is needed in both (larger) offender and general population samples, and with studies comparing offender samples to control groups. Our findings, even though tentative, do show that offender personality characteristics such as optimism, responsibility, empathy, curiosity, and industry, which would seem more favorable to positive intervention outcomes, are related to better cognitive functions. Possible implications could be that interventions in offender populations could be improved by considering both personality dimensions and cognitive proficiencies, tailoring the interventions to individual or specific groups, and not assuming that “one size fits all.” This seems especially relevant in times when demands for cost-effective and efficient interventions are increasing. However, such a conclusion continues to require further research.

## Data Availability Statement

The datasets generated for this study are available on request to the corresponding author.

## Ethics Statement

The DAABS, including the current research questions, was approved by the Regional Research Ethics Committee at Lund University, Dnr 2009/405. The study was performed in accordance with the Helsinki Declaration. All participants consented to participate after having received both written and oral information about the study. Written informed consent was provided by all participants prior to participation. Participants were given 200 SEK (equal to about US$25) to recompense the pay they lost through missed prison activity on the day of assessment. The amount of the monetary reward, small enough to not create an incentive that would compromise the voluntary participating in the study, was approved by the Ethics Committee. Finally, participants who showed signs of severe psychopathology were given the opportunity to be referred to the prison psychiatrist for further assessment and treatment whenever there was such an option.

## Author Contributions

HS, MW, and TN conceived and designed the study and did all the analyses. EB, MW, and BH obtained the data. HS drafted the initial manuscript. Finally, all authors critically revised the manuscript and approved the final version.

## Conflict of Interest

The authors declare that the research was conducted in the absence of any commercial or financial relationships that could be construed as a potential conflict of interest.
